# Clinically relevant anatomical parameters of the replaced right hepatic artery (RRHA)

**DOI:** 10.1007/s00276-015-1491-y

**Published:** 2015-05-17

**Authors:** Grzegorz Staśkiewicz, Kamil Torres, Marta Denisow, Anna Torres, Elżbieta Czekajska-Chehab, Andrzej Drop

**Affiliations:** Department of Human Anatomy, Medical University of Lublin, Jaczewskiego 4, 20-094 Lublin, Poland; Department of Radiology and Nuclear Medicine, Medical University of Lublin, Lublin, Poland; Department of General Surgery, District Specialist Hospital, Lublin, Poland

**Keywords:** Replaced right hepatic artery, Pancreatoduodenectomy, Liver vascularization, Vascular anomalies, Anatomy

## Abstract

**Purpose:**

Vascular anatomy of the liver is subjected to many variations. The most common hepatic artery (HA) replacement is the right hepatic artery (RRHA). Variations of the HA are particularly important consideration when choosing the best surgical procedure or if radiological abdominal intervention is required. In this study, we evaluated the anatomical details of the RRHA origin.

**Methods:**

Retrospective investigation of clinical data from 1569 patients who underwent an abdominal MDCT was performed. The anatomy of RRHA origin was described based on four parameters measured: D—the distance between SMA origin and the RRHA origin, L—the lumen at the place of origin, AH—the origin angle from the SMA in horizontal plane, and AV—the origin angle from the SMA in vertical plane.

**Results:**

RRHA arising from SMA was detected in 10.13 % of cases (159/1569) and its anatomy was subjected to variations. Mean (±SD) of parameters D, L, AH and AV was 27.34 mm ± 6.83, 3.29 mm ± 1.17, 97.27º ± 26.69 and 89.73º ± 20.81, respectively. Values of parameters D and L were significantly higher in males compared to females.

**Conclusion:**

Although radiologists are not always aware of the clinical significance of the RRHA origin, the evaluation of its anatomy is thought to help reduce the risk of inadvertent vascular injury, especially in pancreatoduodenectomy. Detection and evaluation of the RRHA does not necessarily require angio-CT examination. Our study demonstrated that the MDCT, the standard imaging modality for diagnosing the abdominal symptoms, is sufficient to provide the knowledge of the HA abnormalities.

## Introduction

Vascular anomalies in the peripancreatic region include variations of the superior mesenteric artery (SMA), the celiac trunk (CT) or the hepatic artery (HA) [23]. In normal anatomical pattern the liver blood supply involves the common hepatic artery (CHA) arising from the celiac trunk [3, 23]. The CHA gives origin to the proper hepatic artery, which bifurcates into the right and left hepatic arteries (RHA and LHA) [3]. Normal liver vascularization pattern is reported in 50–80 % of cases [1, 9, 17, 23]. The most common HA-replacement found in 13–26 % of cases, is the right hepatic artery (RHA) branching from SMA [9, 11, 29]. Vascular complications form the main cause of morbidity and mortality in patients undergoing pancreatic or duodenal surgery [1, 11]. For that reason, the preoperative information of the vascular system abnormality is particularly important when choosing the most adequate surgical procedure or the radiological endovascular approach [8, 10, 18, 25]. The HA abnormalities are of paramount significance in patients scheduled to liver transplantation or hepatic arterial infusion chemotherapy [4, 13]. The RRHA arising from the SMA emerges as its first branch; as it has a close relationship with the head of the pancreas, running behind or through it, and coursing posteriorly along the common bile duct. These anatomical relations may result in technical difficulties during pancreatoduodenectomy (PD) and they may increase a potential risk of acute haemorrhage or liver ischaemia [13].

The resection of the tumour is the treatment of choice for liver cancer [7]; however, if hepatocellular cancer is identified as unresectable nonsurgical therapies are available, i.e. local ablation, transcatheter arterial chemotherapy (TACE), or combined therapy with TACE followed by radiofrequency thermal ablation [26]. As the vascular anatomy of the liver is subjected to many variations, there is a necessity of recognition concerning any modifications of the hepatic arterial anatomy for successful diagnosis and for the choice of optimal surgical or nonsurgical therapy.

Prior to surgical intervention, the radiological evaluation of the operated area is initially performed using multi-detector computed tomography angiography (MDCT angiography) rather than conventional angiography [5, 22]. The preoperative MDCT angiography can provide the information regarding the vascular system and thus prepare the surgeon to deal with possible intraoperative abbreviations [4].

The study outlines the anatomical details of the right hepatic artery that takes its origin from the superior mesenteric artery. To the best of our knowledge, this is the first description to emphasize the variations existing in the place where RRHA originates.

## Materials and methods

Retrospective investigation of clinical data concerning patients after abdominal MDCT in the Department of Radiology; Clinical Hospital No. 4 in Lublin, Poland, that were performed in 2012. The study consisted of 1569 patients (F:M = 829:740). The mean age of patients was 58.0 years (age range 16–96 years).

Abdominal multi-detector CT exams were performed using a 64-row scanner (LightSpeed, GE), with 64 × 1.25 collimation, slice thickness of 1.25 mm, pitch 1.375:1, at 120 kV current and automatic mA adjustment. During examination 80 ml of non-ionic iodinated contrast was injected into the patients’ antecubital vein.

**Fig. 1:**
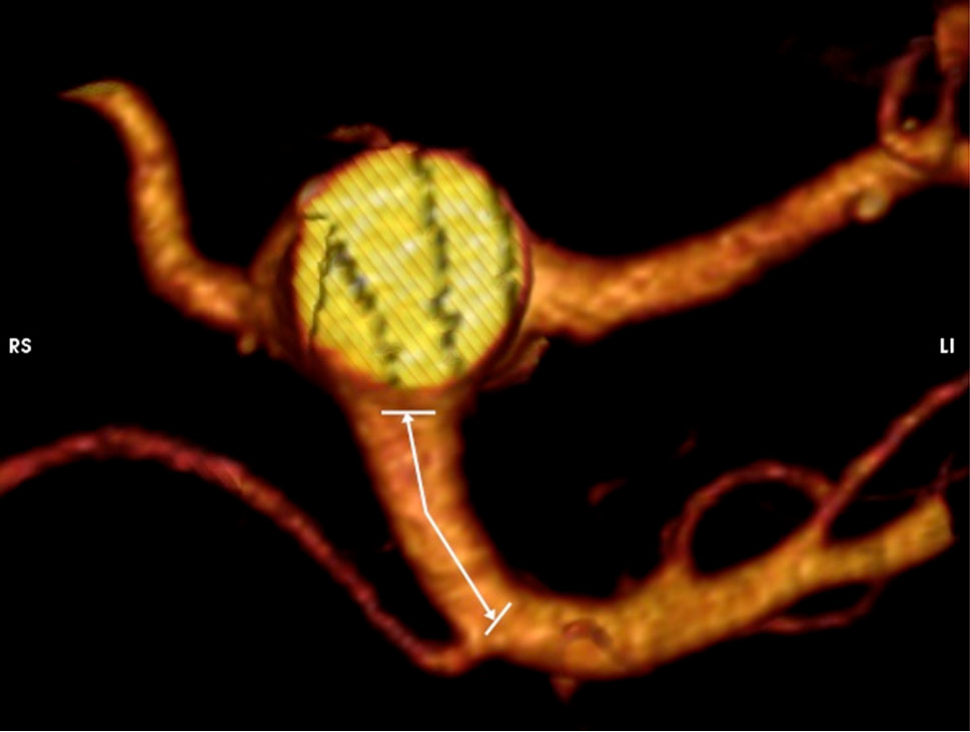
Visualization and measurement method of the parameter D
- Parameter L: the lumen of the RRHA at the place of origin (Fig. 2);

Next, the RRHA cases (*n* = 159) were selected from the examined population. Afterwards, they were analysed using the volume rendering technique. For the more detailed anatomical description of the RRHA origin, four precisely defined parameters were measured:

- Parameter D: the distance between SMA origin from the abdominal aorta and the place of RRHA origin (Fig. 1);

**Fig. 2:**
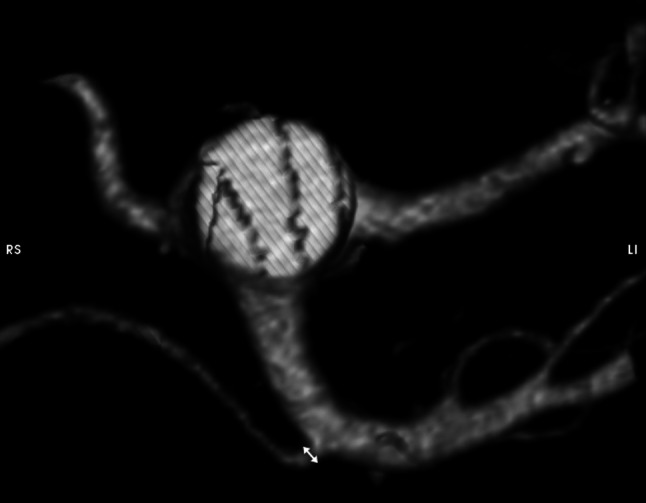
Visualization and measurement method of the parameter L
- Parameter AH: the origin angle of RRHA from the SMA in horizontal plane (Fig. 3);

**Fig. 3:**
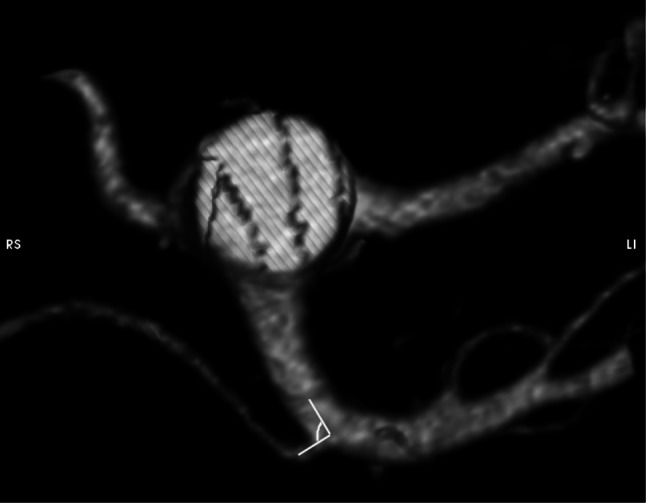
Visualization and measurement method of the parameter AH
- Parameter AV: the origin angle of RRHA from the SMA in vertical plane (Fig. 4).

**Fig. 4:**
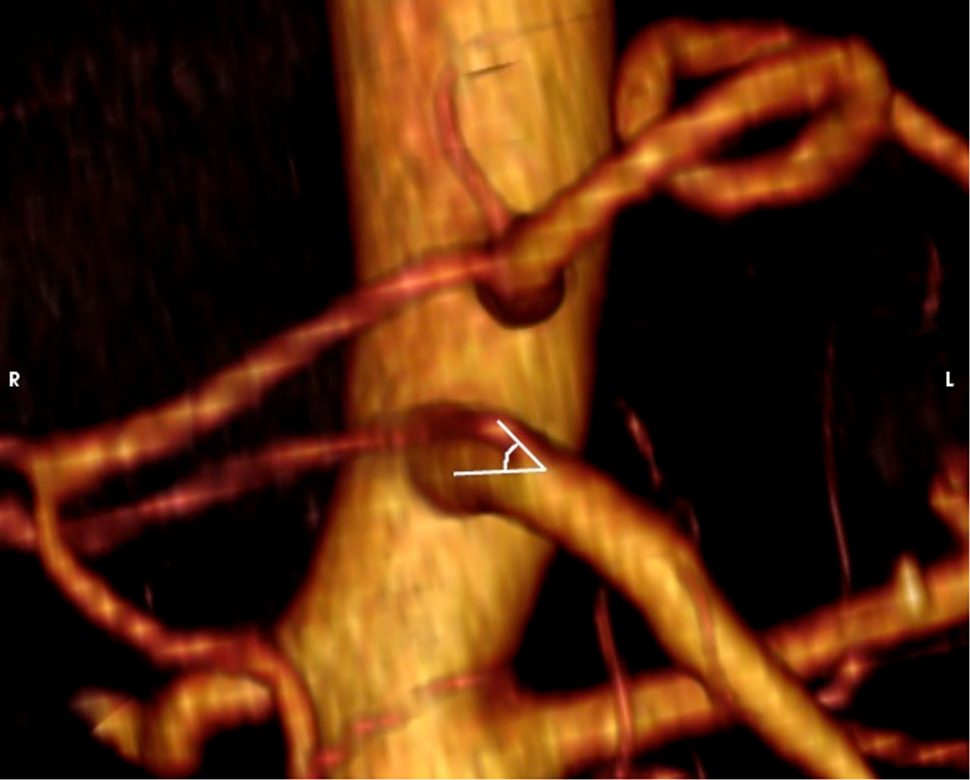
Visualization and measurement method of the parameter AV

**Fig. 5:**
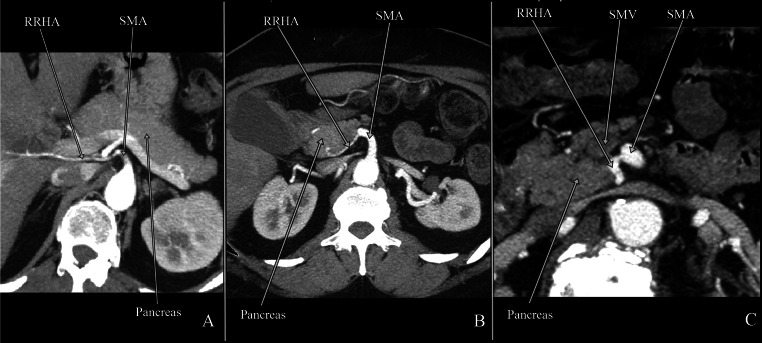
Computed tomography presenting three types of the RRHA. **a** *Type I* RRHA posterolateral to the pancreatic head; **b** *type II* RRHA intrapancreatic (intraparenchymal)—transversing pancreatic head; **c** *type III* RRHA located within superior mesenteric vein (SMV) groove
- Type II: RRHA intrapancreatic (intraparenchymal)—transversing pancreatic head (Fig. 5b)
- Type III: RRHA located within the superior mesenteric vein (SMV) groove (Fig. 5c)

The course of the RRHA in relation to the pancreas was analysed and three types of RRHA were distinguished as described by Jah et al. [10]:

- Type I: RRHA posterolateral to the pancreatic head (Fig. 5a)

Data were presented as mean ± standard deviation (SD) together with minimum and maximum values. The coefficient of variation (CV %) indicating the extent of variability in relation to mean of the population was calculated for the measured parameters. Differences between males and females were analysed using the Mann–Whitney *U* test. Differences of *p* less than 0.05 were considered to be significant. Statistical analysis was performed using SPSS Version 16.0 (SPSS, Inc., Chicago, IL, USA).

## Results

Classical liver vascularization pattern was found in 1410 of 1569 patients (89.87 %). Replaced right hepatic artery (RRHA) arising from the superior mesenteric artery (SMA) was detected in 159 cases (10.13 %).

Each anatomical parameter of RRHA was subject to variation in the studied population. However, the analysis of coefficients of variation suggested that the parameters D and L were more variable than the parameters AH and AL (Table 1).

**Table 1:** Mean values of anatomical parameters of the RRHA origin from the SMA

*n* = 159	Min–max	Mean ± SD	CV (%)
Parameter D (mm)	5.40–48.60	27.34 ± 6.83	25.00
Parameter L (mm)	1.10–6.50	3.29 ± 1.17	35.56
Parameter AH (°)	24.50–156.40	97.27 ± 26.69	27.44
Parameter AV (°)	43.40–160.50	89.73 ± 20.81	23.19

*SD* standard deviation, *CV* coefficient of variation, *D* distance between SMA origin from the abdominal aorta and RRHA origin from the SMA, *L* the RRHA lumen at the place of origin form the SMA, *AH* the RRHA origin angle from the SMA in horizontal plane, *AV* the RRHA origin angle from the SMA in vertical plane

The RRHA arises from the SMA approximately 27.34 mm after its origin from the AA. The distance between SMA origin from the abdominal aorta and the place of RRHA origin (parameter D) was significantly longer in males in comparison to that of females (29.38 mm vs. 25.62 mm). The mean lumen of RRHA in the place of origin (parameter L) was 3.29 mm and was significantly wider in males than in females (3.56 mm vs. 3.07 mm). The RRHA origin angle from the SMA in horizontal (parameter AH) and vertical plane (parameter AV) was 97.27º and 89.73º, respectively.

Anatomical parameters of the RRHA origin compared males and females (Table 2). The statistical differences between male and female groups were observed only for values of D and L parameters. The values of both D and L parameters were significantly higher in males compared to that of females.

**Table 2:** The gender differences impact on the values of anatomical parameters measured in the origin of the RRHA from the SMA

	Females (*n* = 86)		Males (*n* = 73)	
	Mean ± SD	Min–max	Mean ± SD	Min–max
Parameter D (mm)	25.62 ± 6.58	5.40–43.60	29.38 ± 7.00	16.60–48.60
Parameter L (mm)	3.07 ± 1.09	1.20–6.50	3.56 ± 1.21	1.10–6.00
Parameter AH (°)	95.93 ± 29.43	24.50–156.40	98.85 ± 23.15	40.50–148.20
Parameter AV (°)	90.44 ± 20.47	47.90–138.80	88.89 ± 21.32	43.40–160.50

*SD* standard deviation, *CV* coefficient of variation, *D* distance between SMA origin from the abdominal aorta and RRHA origin from the SMA, *L* the RRHA lumen at the place of origin form the SMA, *AH* the RRHA origin angle from the SMA in horizontal plane, *AV* the RRHA origin angle from the SMA in vertical plane

**Fig. 6:**
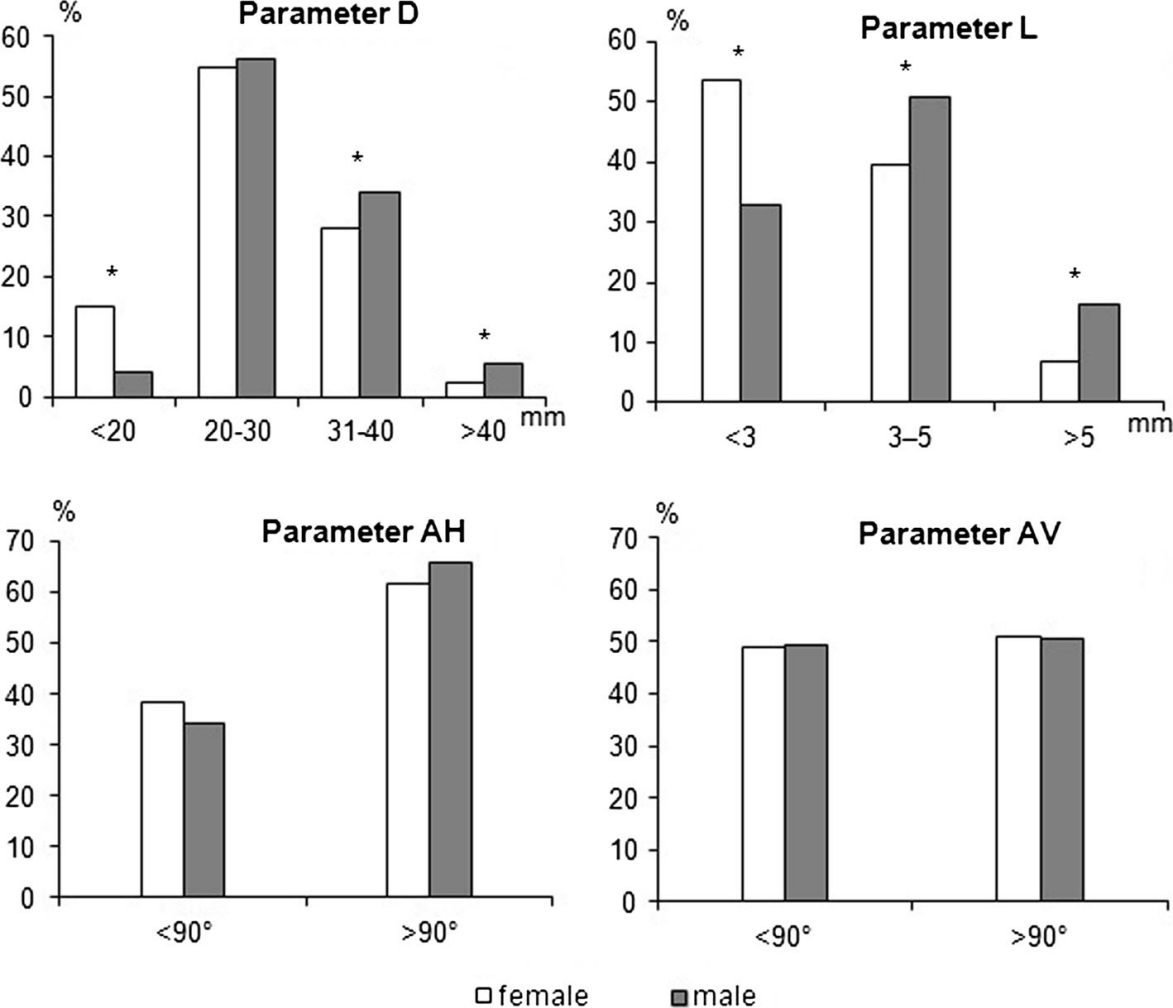
The share of female and male groups in given intervals of anatomical parameters of the origin of the RRHA from the SMA (*n* = 159). *Asterisk* statistically significant differences between males and females in the particular intervals in Mann–Whitney *U* test, *p* < 0.05. *D* distance between SMA origin from the abdominal aorta and RRHA origin from the SMA, *L* the RRHA lumen at the place of origin form the SMA, *AH* the RRHA origin angle from the SMA in horizontal plane, *AV* the RRHA origin angle from the SMA in vertical plane

The distribution of the values of the anatomical parameters of the RRHA origin indicate the most frequent values of the parameter D is between 20 and 30 mm (both for females and males), and for the parameter L is <3 mm for females and between 3 and 5 mm for males (Fig. 6).

Anatomical parameters of the RRHA were evaluated according to the RRHA course in relation to the pancreas (Table 3). Type I was most frequent and observed in 90 % of cases. Types II and III were observed in 6 and 3 %, respectively. There were no statistical differences between the three types of RRHA and anatomical parameters as well as in the RRHA type occurrence between males and females.

**Table 3:** Mean values of anatomical parameters of three types of the RRHA course in relation to the pancreas

	M:F	Parameter D (mm)	Parameter L (mm)	Parameter AH (°)	Parameter AV (°)
		Mean ± SD (min–max)	Mean ± SD (min–max)	mean ± SD (min–max)	mean ± SD (min–max)
Type I	66:77	27.69 ± 6.84 5.40–48.60	3.29 ± 1.16 1.10–6.00	98.37 ± 25.97 24.50–156.50	89.47 ± 21.18 43.40–160.50
Type II	5:6	24.18 ± 5.81 16.30–32.30	3.29 ± 1.45 1.40–6.50	91.46 ± 33.06 43.30–146.20	94.42 ± 19.93 64.40–122.90
Type III	2:3	24.56 ± 7.38 13.60–33.20	3.40 ± 1.16 2.50–5.40	78.68 ± 30.00 31.80–115.20	86.88 ± 11.18 72.30–102.90

*M:F* males:females, *SD* standard deviation, *D* distance between SMA origin from the abdominal aorta and RRHA origin from the SMA, *L* the RRHA lumen at the place of origin form the SMA, *AH* the RRHA origin angle from the SMA in horizontal plane, *AV* the RRHA origin angle from the SMA in vertical plane, *type I* RRHA posterolateral to the pancreatic head, *type II* RRHA intrapancreatic (intraparenchymal)—transversing pancreatic head, *type III* RRHA located within SMV groove

## Discussion

Vascular anatomy of the liver is subject to many variations [21]. The present research of the homogeneous Polish population revealed the occurrence of the right hepatic artery (RRHA) branching from the superior mesenteric artery (SMA) in 10.13 % of cases. The value between 8 and 21 % was shown in previous reports [6, 9, 14, 17, 25], and in each study the RRHA was defined as the most common vascularization abnormality of the hepatic artery (HA). Frequency of the hepatic arterial variations in the peripancreatic region indicates the necessity of the preoperative assessment of the RRHA anatomy. Despite the fact that abnormality often appears, the detailed measurements of anatomical parameters of RRHA origin are not available in literature reports. Therefore, we precisely defined parameters of RRHA anatomy. We revealed the variability in the anatomy of RRHA and gender impact on the two out of four parameters measured. Preoperative recognition of RRHA anatomy should help to avoid unexpected complication during surgeries performed in the pancreato-duodenal region and during surgical and endovascular treatments of the liver.

RRHA usually passes lateral and behind the portal vein and joins the structures of the portal triad when entering the hepatoduodenal ligament posterolateral to the common bile duct. At this point, palpation can be used in the epiploic (Winslow) foramen [2].

The vascular anatomy of the operated region is highly required during pancreatoduodenectomy (PD). Typically, the kocherization of the duodenum and opening of the pars flaccida of the lesser omentum is followed by the palpation of the proper hepatic artery [29]. The additional pulsation present in the left lateral border of the hepatoduodenal ligament may be synonymous with the arterial abnormality. In the case of the RRHA, the pulsation is possible behind the pancreatic head or higher posteriorly to the portal vein and the common bile duct [29]. However, the RRHA from the SMA can course within or along the ventral side of the pancreas. The RRHA passing through the pancreatic parenchyma was reported in 50 %, whereas the other 50 % runs behind the pancreas [16, 30]. In our study, in 90 % of cases the RRHA courses posterolateral to the pancreatic head. The RRHA coursing through the pancreatic parenchyma was observed in 7 % of cases, while RRHA located in the groove of the SMV was uncovered in 3 % of cases. Additionally, the distance between SMA origin and the place of RRHA origin longer than 40 mm occurred with the lowest frequency, and results in close RRHA course to the pancreas. Such topography develops awareness of arterial injuries during intervention in the peripancreatic region. However, when the recognition is possible a complication can be avoided.

The evaluation of the RRHA anatomy is also important during liver transplantation, the therapy has progressively increased in numbers over the last decade [15]. Frequency of arterial hepatic variations is reported at the level of 15 % of donor livers [9, 13]. In the living donor liver transplantation (LDLT), injuries to the RRHA may lead to a reduction in the functional volume of the right hepatic lobe and can also result in the decrease of the blood supply in both right lobe and the gallbladder. Complications associated with the RRHA injuries can be severe and may include insufficient hepatic volume, ischaemic cholangiopathy or hepatic artery thrombosis [27].

Moreover, vascular anomalies are reported to increase the rate of morbidity and mortality during PD, as they adversely affect the outcomes of patients [31]. Additionally, vascular abnormalities are associated with an increased risk of disease recurrence, especially when the RRHA injury resulted from radical LN dissection [28]. Kim et al. reported poor outcomes of patients with the RRHA describing an accidental portal vein injury and transection of the RRHA [12]. Furthermore, numerous evidences suggest that complications pertained to the liver or the bile duct developed during the RRHA injury have serious consequences, e.g. may induce a leakage in the bilioenteric anastomosis [2, 24].

It is crucial to define the model of hepatic arteries for surgeons, as well as for diagnostic and interventional radiologists. The pattern of hepatic arteries is significant in the treatment of hepatocellular cancers identified at the initial stage as the cancer does not yet qualify for the transplant waiting list [26]. If the transplantation is not possible the transarterial therapy with the transcatheter arterial chemoembolization or transarterial radioactive iodine with lipiodol as the most commonly used technique is one of the treatment options [20].

In this study, the origin angle of RRHA from the SMA in vertical plane lower than 90° was observed with frequency of 36.5 %. In this group of patients, the acute angle makes the accessibility to the RRHA very difficult. Therefore, additional evaluation of the distance between SMA origin and RRHA origin may help to recognize where the RRHA origin from the SMA can be expected.

Preoperative recognition of abdominal vascular architecture in the peripancreatic region provides valuable information that can help to choose the most appropriate strategy during surgery. Commonly performed radiological examinations include CT angiography or MDCT and MRCA, as a routine preoperative examination in case of potential radical resection for pancreatic cancers [19]. Additionally, MDCT is proposed as a satisfactory examination for detection of hepatic arteries patterns [14].

## Conclusion

The report revealed that the anatomy of origin of RRHA, the most common anomaly of the hepatic arteries, is subject to considerable variations. The findings are relevant for radiologists responsible for providing accurate anatomical information and for surgeons involved in the treatment of pathologies in the upper abdomen. It was shown that preoperative MDCT routinely performed could successfully be used to detect and evaluate the RRHA.
